# Biosimilar agents in oncology/haematology: from approval to practice

**DOI:** 10.1111/j.1600-0609.2010.01566.x

**Published:** 2011-04

**Authors:** Dietger Niederwieser, Stephan Schmitz

**Affiliations:** 1 Department of Hämatologie/Onkologie/Hämostaseologie, Universitätsklinikum LeipzigLeipzig, Germany; 2 Onkologische SchwerpunktpraxisKöln, Germany

**Keywords:** Biosimilars, granulocyte colony-stimulating factor, biologics, substitution, traceability, extrapolation, international non-proprietary names

## Abstract

The regulation of biosimilars is a process that is still developing. In Europe, guidance regarding the approval and use of biosimilars has evolved with the products under consideration. It is now more than 3 years since the first biosimilar agents in oncology support, erythropoiesis-stimulating agents, were approved in the EU. More recently, biosimilar granulocyte colony-stimulating factors have received marketing approval in Europe. This review considers general issues surrounding the introduction of biosimilars and highlights current specific issues pertinent to their use in clinical practice in oncology. Information on marketing approval, extrapolation, labelling, substitution, immunogenicity and traceability of each biosimilar product is important, especially in oncology where patients are treated in repeated therapy courses, often with complicated protocols, and where biosimilars are not used as a unique therapy for replacement of e.g. growth hormone or insulin. While future developments in the regulation of biosimilars will need to address multiple issues, in the interim physicians should remain aware of the inherent differences between biosimilar and innovator products.

## First-generation biologics and ‘biosimilars’: both are unique molecules

**Figure 1:**
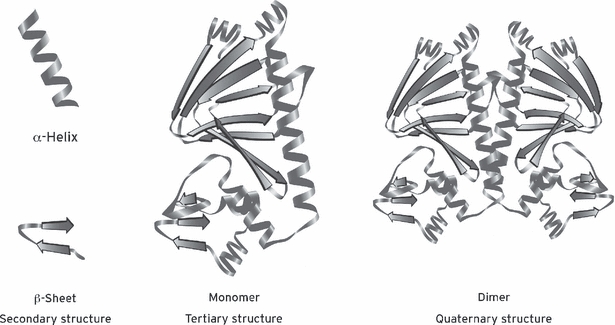
Secondary, tertiary and quaternary structures of protein drugs. Adapted from: Krämer I & Jelkmann W. 2008 (92)

Recombinant biologic agents are proteins or peptides, often similar to endogenous hormones, cytokines or antibodies, derived using DNA technology (1). These proteins fold into complex molecules whose architecture is a key determinant of their function (Fig. 1) (2, 3). The average molecular weight of a biologic ranges from 4000 Daltons (Da) for non-glycosylated proteins to > 140 000 Da for monoclonal antibodies (4) and is much greater than that of small molecule chemical pharmaceuticals, whose average molecular weight ranges from ∼ 160 to 800 Da (2). Recombinant biologic agents are produced from cultured, genetically modified cell lines and extracted through complex and lengthy purification procedures (2). As a consequence of their complexity and cell-based production, biologic agents are inherently more difficult to characterise than standard chemically derived agents (2, 3, 5). The properties of biologic agents are dependent on their manufacturing process, and even minor alterations at any one of the numerous stages of production have the potential to influence the end product (1–3). (See Mellstedt *et al.* (3) for an evaluation of the steps involved in the manufacture of biologics).

Historically, exclusivity expiry of standard small molecule pharmaceutical agents has seen the development of generic versions, which are exact copies of the innovator product. Expiry of patents and data protection on first-generation biologics has, however, brought about a new situation; for the reasons discussed earlier, developing an exact copy of a biologic agent is technically impossible (1–3, 5, 6). For example, a ‘follow-on’ biologic agent will not be manufactured using an identical process to the innovator product, as this is proprietary knowledge (3, 7). (See Kuhlmann and Covic (2) for a more detailed discussion of the protein science of biologics). These ‘follow-on’ biologics are therefore unique molecules, rather than identical generic copies of innovator biologics, and should be considered as such (3). The European Medicines Agency (EMA) recognised this situation, stating that –‘*due to the complexity of biological/biotechnology-derived products the generic approach is scientifically not appropriate for these products’* (5) – hence a new regulatory pathway was needed. The term ‘biosimilar’ was coined to refer to a product that is similar, but not identical, to the innovator biologic product (8).

Previous authors have reviewed the manufacturing and approval process for biosimilars, speculating on what issues might arise once such agents are introduced (1, 3, 9, 10). It is now 3 years since the first biosimilars were approved for use in Europe in the oncology/haematology setting. Such agents have increased the prescribing options open to physicians with regard to biologic medicines. In this article, we seek to make physicians aware of the general ongoing developments surrounding biosimilars and to highlight specific issues that are pertinent to their use in oncology clinical practice. The EMA states that the decision to treat a patient with an innovator or biosimilar medicine should be taken by a qualified healthcare professional (8). Our intention is not to discourage physicians from considering the use of biosimilar products, but to highlight that they need to be informed on biosimilar products with regard to marketing authorisation, extrapolation, labelling, substitution and pharmacovigilance – in order to avoid complications and problems associated with this new product class in general and, more specifically, in oncology.

## The regulation of biosimilars is an evolving process

**Figure 2:**
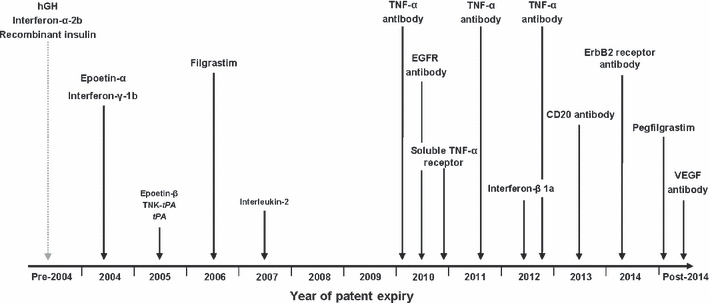
Patent expiry for innovator biologic medicines in the EU. Source: Schellekens H *et al.*, 2005 (1); Ledford H *et al.*, 2007 (93)

The European Union (EU) has led the way in the regulation of biosimilars, responding to the patent and data protection expiry of several innovator biologic medicines in recent years (Fig. 2). The approval of ‘biosimilars’ by regulatory bodies and coordinating authorities is a process that is still evolving – both in the EU and around the world.

### European Medicines Agency

The EMA has established the first regulatory framework for assessing biosimilars. This is distinct from the process required for generics (11, 12) and less extensive than the process required for registration of a new biologic product (13–16) or a new chemical product.

An overarching guideline defines the concept of biosimilars and sets out the ‘comparability exercise’ through which similarity between a biosimilar product and its reference innovator product must be demonstrated in order to gain regulatory approval (5). The reference product must be an innovator product, which is already authorised in the EU, with a similar active substance. Pharmaceutical form, strength and route of administration should be the same as that of the reference product (5). Comparability must be demonstrated in terms of quality, efficacy and safety (17,18). Comparability of quality is assessed for the active substance and the finished medicinal product and must be demonstrated for analytical methods, physico-chemical characterisation, biological activity and purity of the similar biologic medicinal product (17). Comparability of efficacy is assessed via non-clinical comparative *in vitro* and *in vivo* studies, and a repeat-dose toxicology study of sufficient length to allow detection of relevant differences in toxicity (18). Comparable clinical efficacy is evaluated through a stepwise procedure beginning with clinical pharmacokinetic (PK) and pharmacodynamic (PD) studies, followed by 2- or 3-arm clinical efficacy studies; in certain cases, PK/PD studies alone may suffice (18). Finally, clinical safety should be evaluated in comparative clinical studies assessing the adverse event profile and immunogenicity. Plans for postmarketing surveillance – pharmacovigilance and risk management – should be provided (18).

The data requirements and studies necessary to demonstrate comparability differ between product classes and are laid out in specific guidelines for somatropin (19), human soluble insulin (20), interferon alpha (21), erythropoietins (22), granulocyte colony-stimulating factor (23), and most recently for biosimilar low molecular weight heparins (24). Product classes currently under consideration for specific guidelines include recombinant follicle stimulation hormone, recombinant interferon beta and monoclonal antibodies (25).

### Food and Drug Administration

In March 2010, the US Congress passed legislation creating a legal pathway for biosimilars under the Patient Protection and Affordable Care Act, as part of the wider healthcare reform legislation (26). The legislation providing an approval pathway for biosimilar biological products is outlined in section ‘Title VII – Improving Access to Innovative Medical Therapies: Subtitle A – Biologics Price Competition and Innovation’. Biosimilarity is established where the biological product is highly similar to its reference product, notwithstanding minor differences in clinically inactive components, and there are no clinically meaningful differences between the biological product and the reference product in terms of the safety, purity and potency of the product. The Food and Drug Administration (FDA) will be responsible for reviewing applications for biosimilarity.

### World Health Organization

The World Health Organization (WHO) has also recognised that the approach established for generic medicines is not suitable for development, evaluation and licensing of similar biotherapeutic products (SBPs) (27). In April 2010, the WHO Expert Committee on Biological Standardization published final ‘Guidelines on Evaluation of SBPs’, as part of its mandate to assure global quality, safety and efficacy of biotherapeutics (27). Similar to the EMA, the WHO advocates a stepwise approach for the licensing of an SBP that depends on demonstrated similarity in quality, non-clinical and clinical parameters to a suitable reference biotherapeutic product (RBP). The RBP must be an innovator product of similar active substance, with the same dosage form and route of administration, licensed on the basis of a full registration dossier.

The comparability exercise between the SBP and the RBP in the quality part represents an additional element to the ‘traditional’ full quality dossier. Non-clinical evaluation of new biotherapeutics normally encompasses a spectrum of PD, PK and toxicological studies. Clinical evaluation is also via a stepwise procedure, beginning with PK and PD studies followed by pivotal clinical trials, although in certain cases comparative PK/PD studies may suffice. Similar efficacy (equivalence designs) will usually have to be demonstrated; however, non-inferior study design may be considered if appropriately justified. Safety data should be obtained in a sufficient number of patients, preferably in a comparative design. Prelicensing safety data obtained from clinical trials can be expected to detect frequent and short-term adverse events/reactions; however, additional postmarketing monitoring of an SBP will be necessary. Immunogenicity should be investigated preauthorisation in humans.

Extrapolation of efficacy and safety data to other indications may be possible if certain prerequisites are met, e.g. the clinically relevant mode of action and/or involved receptor(s) are the same, and no unique/additional safety issues are expected for the extrapolated indication(s).

The WHO is also the coordinating authority responsible for assigning international non-proprietary names (INN) to identify pharmaceutical substances (28). In September 2006, the WHO recommended against introducing distinctive INNs to indicate a biosimilar product, but acknowledged that INNs should not be relied upon as the only means of product identification for biologicals nor as the sole indicator of product interchangeability (29). This has been incorporated into current guidelines and the WHO recognises that National Regulatory Authorities (NRA) need to define interchangeability and substitution of RBP with SBP and labelling and prescribing information. The WHO recommends that the SBP should be clearly identifiable by a unique brand name, which should be stated alongside the INN (27). Furthermore, provision of the lot number is essential and critical for traceability in cases where problems are encountered. Prescribing information should be as similar as possible to that of the RBP, except for product-specific aspects, and if the RBP has fewer indications related text may be omitted. The NRA may choose to mention the SBP nature of the product and the studies that have been performed with the SBP and/or to include instructions for the prescribing physician on how to use SBP products (27).

### Other regulatory agencies in the world

In June 2006, the Australian Therapeutic Goods Administration adopted the European guidelines for registration and approval of biosimilars – allowing for the registration of a biosimilar medicine on the basis of the evaluation of an abbreviated quality and clinical dossier (30). In the middle and near East, ongoing discussions have utilised EMA guidelines as the basis for recommendations (31). The same is true of Canada, who in March 2010 published revised submission requirements for ‘subsequent entry biologics’ (SEB) that largely follow EMA guidelines (32). Non-Canadian-licensed innovator products may also constitute the reference product, providing that the sponsor demonstrates a link to a biologic drug authorised in Canada to which the SEB will be subsequent (33). The Japanese Ministry of Health, Labour and Welfare issued guidelines for the approval of biosimilars in March 2009 (34). This process is separate to that for conventional chemical generic drugs, with a review process and data requirements more akin to those for new drugs (34, 35). Biosimilar products should be clearly identified by brand name, and non-proprietary names should be followed by ‘kozoku-1’, meaning ‘follow-on-1’, and so on (34, 35). In October 2009, Japan approved a somatropin human growth factor biosimilar (36).

## Oncology/haematology biosimilars approved in Europe

### Erythropoiesis-stimulating agents

Erythropoiesis-stimulating agents (ESAs) up-regulate red blood cell production and are indicated for the treatment of symptomatic anaemia in adult cancer patients with non-myeloid malignancies receiving chemotherapy. Two epoetin alfa (recombinant erythropoietin) products (Epogen® (Amgen, Thousand Oaks, CA, USA) and Procrit® (Centocor Ortho Biotech, Horsham, PA, USA)) received marketing approval in the United States in 1989. A third innovator epoetin alfa product Erypo®/Eprex® (Janssen-Cilag GmbH; Baar, Switzerland) is approved in Europe; ESAs are among the most widely used biologics (4). Two biosimilar epoetins were the first ‘oncology’ biosimilars to receive European marketing approval utilising the ‘Similar Biological Medicinal Product’ application (5). In both cases, the comparability exercise was performed in patients with anaemia associated with chronic renal failure, using epoetin alfa (Eprex®) as the reference product. Supportive data from single-arm studies in patients with CIA were supplied for both products. It is particularly interesting to note that the data presented for approval in each of these two cases varied because of the rapidly evolving procedures for biosimilar approval during this period. Clinical PD data were not included in the dossier presented for SB309 epoetin zeta (37, 38), as this was not required under guidelines at the time; whereas PD data from healthy volunteers formed part of the comparability exercise for the approval of HX575 epoetin alfa (39–41). SB309 epoetin zeta and HX575 epoetin alfa are single molecules licensed to multiple marketing authorisation holders and marketed under several different names (Table 1).

**Table 1:** Overview of oncology/haematology biosimilars licensed in Europe

Molecule	INN	Brand name
Biosimilar erythropoietins		
HX575	Epoetin alfa^1^	Abseamed® (39)
		Binocrit® (40)
		Epoetin alfa Hexal®(41)
SB309	Epoetin zeta^2^	Retacrit® (37)
		Silapo® (38)
Biosimilar G-CSFs		
XM02	Filgrastim^3^	Tevagrastim® (43)
		Ratiograstim® (44)
		Filgrastim ratiopharm® (46)
		Biograstim® (45)
EP2006	Filgrastim^4^	Zarzio® (47)
		Filgrastim Hexal® (48)
PLD108	Filgrastim	Nivestim® (49)

1 Single molecule HX575 licensed to multiple marketing authorisation (MAA) holders.

2 Single molecule SB309 licensed to multiple MAA holders.

3 Single molecule XM02 licensed to multiple MAA holders.

4 Single molecule EP2006 licensed to multiple MAA holders.

G-CSF, granulocyte colony-stimulating factor; INN, international non-proprietary name.

A recent assessment of the similarity of SB309 highlighted necessary caveats in the assessment of similarity in biosimilars (42). The EMA recommends that similarity of potency to the innovator product is established within acceptable limits, for example those defined by the European Pharmacopeia as 80–125% (error limits 64–156%) for an *in vivo* bioassay. Thus, despite satisfying this requirement, differences in potency of biological products are probable. In the case of SB309, bioactivity (hence potency) has been shown to be ∼10% lower compared to the reference product epoetin alfa (Eprex®) in patients with renal anaemia (42).

### Granulocyte colony-stimulating factor

More recently, granulocyte colony-stimulating factor (G-CSF) filgrastim biosimilars have received approval; XM02 in September 2008 (43–46), EP2006 in February 2009 (47, 48) and PLD108 in June 2010 (49). Filgrastim is a widely used biologic, over 7.7 million patients have been exposed to the innovator product Neupogen® (Amgen, Thousand Oaks, CA, USA) since it received marketing approval in 1991 (50). In the EU, filgrastim is indicated in adults and children to shorten the duration of neutropenia and reduce the incidence of febrile neutropenia following receipt of cytotoxic chemotherapy (51). It is also used to aid delivery of chemotherapy to maintain dose intensity and to support dose-dense chemotherapy (51, 52). Filgrastim is also indicated to mobilise peripheral blood progenitor cells (PBPC) in both cancer patients and healthy donors and to support engraftment and neutrophil recovery after stem cell transplantation (51). Outside the oncology setting, filgrastim is indicated for the treatment of severe chronic neutropenia (51, 53, 54) and to maintain neutrophil counts or reverse neutropenia in patients infected with human immunodeficiency virus (51).

The comparability exercise for approval of the biosimilar filgrastim products XM02, EP2006 and PLD108 was conducted using filgrastim (Neupogen®) as the reference product (Table 1). XM02 is a single molecule licensed to multiple marketing authorisation holders and marketed under several different names (43–46). In accordance with EMA guidelines, comparability was assessed in a single indication for which Neupogen® is approved for the reduction of chemotherapy-induced neutropenia (CIN). Efficacy was assessed in a comparative study in breast cancer patients at high risk of CIN, and supportive studies provided safety data from CIN patients with lung cancer and non-Hodgkin lymphoma. The biosimilar filgrastim EP2006 is also a single molecule licensed to two marketing authorisation holders and marketed under different names (47, 48). In contrast to XM02, the comparable efficacy of EP2006 was established on the basis of PK and PD studies in healthy adults, with a single-arm, non-comparative study in patients at high risk of CIN with breast cancer providing supportive safety data. PLD108 is a single molecule licensed to a single marketing authorisation holder (49). Comparability with the reference product filgrastim (Neupogen®) was assessed in breast cancer patients at high risk of CIN.

## Biosimilars in oncology practice

Previous reports on biosimilars raised several issues surrounding their introduction into clinical practice (3, 9, 10, 55). Given that biosimilar agents are now approved in the EU, these issues can be discussed more comprehensively on the basis of published data and regulatory documents. Issues specific to the introduction of the first biosimilar ESAs have been reviewed elsewhere (7). We would like to focus on biosimilars in oncology practice, where they are not used simply for the replacement of hormones (e.g. growth hormones, insulin) or the treatment of renal insufficiency (i.e. erythropoietin); but as supportive therapy for immunosuppressed patients receiving multiple cycles of cytotoxic therapy, or for healthy stem cell donors who obtain no direct therapeutic benefit from treatment.

In general, oncologists should be aware that the terms ‘biosimilar’, ‘similar biotherapeutic product’, ‘subsequent entry biologic’ or ‘follow-on biologic product’ refer to the same type of product. Furthermore, it is important to have a detailed knowledge of the characteristics of these products, including extrapolation, substitution, labelling, traceability, safety and immunogenicity. In the following sections, we will give an overview of these key points for each biosimilar product.

### Extrapolation of indication in the EU

Extrapolation involves the approval of a drug for indications for which it has not been evaluated in clinical trials (3). For the filgrastim biosimilars XM02, EP2006 and PLD108, extrapolation from data in healthy adults and CIN has allowed approval in all indications of the reference product (18, 23, 43–49). Although fully compliant with current guidelines, extrapolation of data from one indication to another has raised some concerns, particularly with regard to the use of biosimilar filgrastim for PBPC mobilisation and transplantation (56). In the European Public Assessment Reports (EPARs) for XM02, PBPC mobilisation was highlighted by the EMA as an ‘area of uncertainty’, because it is not known whether efficacy in CIN can be fully extrapolated to PBPC mobilisation (43–46). Following discussions with the EMA, XM02 was approved with routine pharmacovigilance for PBPC mobilisation (43–46). The risk-management plan for EP2006 specified additional follow-up of healthy adults who participated in a phase I study and 5-year follow-up of healthy stem cell donors in cooperation with aphaeresis centres (47, 48). Similarly, potential risks to healthy stem cell donors were acknowledged in postapproval commitments for PLD108, which included plans for targeted questionnaires and long-term data collection, in addition to routine pharmacovigilance (49). The European Group for Blood and Bone Marrow Transplantation, however, advised against use of biosimilar G-CSFs in unrelated healthy stem cell donors until efficacy and safety data have been collected in clinical trials in the autologous setting, encompassing an adequate number of stem cell mobilisation procedures with adequate follow-up (57).

No experience concerning extrapolation to special patient populations has been reported, as the biosimilar filgrastim products XM02, EP2006, and PLD108 have not been administered to children, patients with renal or hepatic insufficiency or patients with acute myeloid leukaemia (43–49).

### Substitution in the EU

Substitution of one product with another that has the same INN, by the pharmacist, is common practice with generic drugs, but is not appropriate with biologics. This has been clarified by several European institutions and agencies, including the EMA, which advises that the decision to treat a patient with a reference or biosimilar medicine should be taken following the opinion of a qualified healthcare professional (8). As a consequence of their complexity, automatic substitution of biologics could give rise to different clinical consequences and should be ruled out for reasons of patient safety (9, 58).

Measures to prevent automatic substitution (dispensing of generic drugs in place of prescribed innovator products by pharmacists without the knowledge or consent of the treating physician (3)) are already in place in several European countries, and other countries have taken steps to limit or prohibit substitution of innovators with biosimilars (Table 2). Substitution is also the subject of debate in other regions: in July 2010 Health Canada stated that it does not support automatic substitution of an SEB for its reference biologic drug as differences in manufacturing over time may lead to changes that affect drug products; Health Canada (59) therefore recommends that physicians make only well-informed decisions regarding therapeutic interchange. In the Middle East, it has been recommended that products should be clearly identified as biosimilars on the label (31).

**Table 2:** Some EU countries which have taken specific measures to limit or prohibit substitution of innovators with biosimilars, in others current law prohibits automatic substitution of innovators with generics

Country, Regulation (Year regulation came into force)		Specific to biologics?
No automatic substitution allowed		
France 2006	In 2006 French Law (LOI no 2006-3062, article 11) prohibited automatic substitution of biosimilar products (75).	Yes
Germany 2008	The automatic substitution of biologics is not permitted in Germany. In January 2008, German Social Law (Rahmenvertrag 20080117, § 129) indicated that pharmacists are obliged to prescribe a generic product when available, and that physicians must actively prohibit automatic substitution when prescribing, however, this does not apply to biologics (76)	No
Greece 1976 & 1993	Greek Law (ND 96/1973 – Article 13, section 3) states that pharmacists are obliged to provide the exact pharmaceutical products mentioned in a medical prescription and are absolutely prohibited from substituting them with other pharmaceutical products (77).	No
	This is reinforced by the Greek Code of Ethics for Pharmacists (PD 340/1993 - Article 23), which states that pharmacists are not at liberty to substitute the pharmaceutical products stated in a prescription with any other product (78).	
Italy 2007	Based on a note from the Ministry of Health, the Italian Council of State issued opinion (n.3992.07) stating that biosimilars cannot be substituted (79).	Yes
Slovenia 2008	Slovenian Medical Society guidelines prohibit the substitution of biologics, any medicinal product should be approved for substitution by the Slovenian Medical Society (80).	Yes
Spain 2007	In 2007, the Spanish Health Agency (Ministerio De Sanidad Y Consumo) stated that biologics as not substitutable - ORDEN SCO/2874/2007 (81).	Yes
Sweden 2007	In 2007, the Swedish Medicines Agency (MPA) issued a statement saying that biologics are not interchangeable and are not recommended for substitution (82).	Yes
UK 2010 (ongoing)	At present there is no automatic substitution of biologics in the United Kingdom, if the physician prescribes by brand, this is what must be given. There is ongoing consultation about the introduction of automatic substitution. The Department of Health (DoH) and the Association of the British Pharmaceutical Industry (ABPI) have proposed to Medicines and Healthcare products Regulatory Agency (MHRA) that biologics/biosimilars should be exempt from automatic substitution and that biologics should only be substituted with prescribing physician's knowledge and prior consent. The MHRA has stated that it is best practice to prescribe by brand name to ensure traceability (83).	Yes
Automatic substitution must be actively prohibited by the physician		
Czech Republic 2008	In January 2008, Czech Drug Law (No 378/2007, § 83, article 2) was updated to state that automatic substitution of any originator product with a generic must be actively prohibited by the physician (84).	No
Official list stating which products cannot be substituted		
Denmark 2010	Biosimilars can be substituted for each other, but not for reference products in the substitution lists issued by the Danish Medicines Agency (DKMA; (85)).	Yes
Finland 2009	The Finnish Regulatory Agency (FINMEA) states that products given parenterally are not substitutable (86).	No
Hungary 2009	Biosimilar products are absent from the positive substitution lists issued by the Hungarian National Institute of Pharmacy, thereby preventing their automatic substitution (87).	Yes
Norway 2010	In Norway, all pharmaceuticals that are regarded as generics or therapeutically equivalent should be put on an automatic substitution list. Although filgrastim was initially considered for substitution, in July 2010 the Norwegian Medicines Agency (NOMA) announced that until further notice filgrastim will be taken off the substitution list (88). Biosimilars are absent from the October 2010 substitution lists (89).	Yes
Slovakia 2008	Biosimilar products are absent from positive substitution lists published by The Slovakian Ministry of Health (90).	
Physicians obliged to prescribe by brand name		
Austria 2005	Austrian Medicines Law (AMG § 10 section 8) recognises that biosimilars are not generics. Physicians are obliged to prescribe by brand name and to look for the cheapest but best medicines for their patients therefore there is no obligation to substitute biologics and this responsibility lies with the physician (ökonomische Verschreibung, RÖF 2005; (91)).	No

### Labelling

In order to maintain current standards of patient safety regarding the use of biologic agents, it has been suggested that distinct brand names, together with an adapted summary of product characteristics (SmPC), are used to identify both innovator and biosimilar agents (6). Both biosimilar ESAs and G-CSFs have distinct brand names. The SmPC for biosimilar epoetin alfa and epoetin zeta include data from the reference product Eprex® SmPC; no biosimilar data are provided and, except for mention of the brand name, it is not clear that the product being described is a biosimilar (60–64). The SmPC for the biosimilar filgrastim products XM02, EP2006, and PLD108 present data from the SmPC of the reference product Neupogen®. Comparability studies to a ‘reference product’ are mentioned, giving some indication that the product being described is a biosimilar; however, biosimilar data are not presented and extrapolated indications are not identified as such (65–71). Healthcare professionals who are unfamiliar with the regulatory process for biosimilars may not be aware that the majority of the product information presented is not derived from the product under consideration.

### Traceability

The traceability of biologics, including biosimilars, is important, as all these products have differences and biosimilars are not identical to innovators. The exact product prescribed should therefore be identified and identifiable to enable accurate pharmacovigilance (6). In line with several other national regulatory authorities (30,31,35) and the WHO (27), the EMA requests that the specific medicinal product given to the patient should be clearly identified (5). In oncology/haematology, the biosimilar G-CSFs have the INN filgrastim, the same as the innovator product (43–48, 51). In contrast, biosimilar ESAs that used the same reference innovator product (Eprex® epoetin alfa) have been assigned two different INNs – with one product receiving the same INN as the innovator (39–41) and another being assigned a different INN epoetin zeta (37, 38).

### Safety and immunogenicity

Immunogenicity is the most important safety issue concerning all biosimilar products (1, 72, 73). Analytical tests and clinical trials detect many, but not all, potential immunogenic responses; so postmarketing commitments and pharmacovigilance are critical (1, 73). In oncology/haematology, biosimilar ESAs have additional postmarketing studies in their risk-management plan to address safety concerns such as pure red cell aplasia, thrombotic vascular events and tumour growth potential, as well as to monitor potential off-label subcutaneous use in renal anaemia patients (37–41).

In contrast, the postmarketing programme for biosimilar G-CSFs differs between products. Routine risk management and a signal detection procedure for immunologic events are proposed for the biosimilar filgrastim XM02, although this is the first product for which extrapolated indications were granted (43–46). This risk-management plan was approved on the basis that immunogenicity data from comparative clinical trials indicated no significant group differences between cancer patients treated with biosimilar filgrastim and patients treated with the innovator reference filgrastim (Neupogen®). Length of follow-up is important when assessing immunogenicity; however, the duration of follow-up for patients and healthy volunteers who received XM02 is not clearly stated in the product EPARs (23). For the biosimilar filgrastim EP2006, a more extensive postmarketing programme is described, including a phase IV study, and cooperation with the severe chronic neutropenia registry, as well as aphaeresis centres to investigate its use for mobilisation in healthy stem cell donors (47, 48). Some safety and potential immunogenicity differences between the biosimilar filgrastim PLD108 and its reference product (Neupogen®) were reported in the product EPAR (49). A higher incidence of bone pain and myalgia was observed with PLD108, this is addressed in the product label where bone pain is described as ‘common’ for the reference product and ‘very common’ for PLD108 (71). As a potential higher risk of immunogenicity in individuals treated with PLD108 could not be excluded (a low number of patients treated with PLD108 had G-CSF antibodies), the risk-management programme proposed includes plans for targeted questionnaire follow-up of potential immunogenicity in addition to routine pharmacovigilance. Other postapproval commitments for PLD108 include targeted follow-up through the severe chronic neutropenia registry, specialised follow-up for long-term data and cooperation with international transplant centres.

In the United Kingdom, the Medicines and Healthcare products Regulatory Agency have marked the biosimilar ESA products epoetin alfa and epoetin zeta, and the biosimilar filgrastim products XM02, EP2006 and PLD-108 with a black triangle, which indicates these products should be intensively monitored in order to confirm their risk/benefit profile (74). It should be noted that this scheme is not limited to biologics, but applies to all new medicinal products for which limited safety data raises concerns; such triangles would not be applied to a standard generic.

## Summary and outlook

The introduction of biosimilars is a new development; because of inherent differences between biosimilars and innovator compounds, biosimilars undergo more thorough investigation than generic small molecule pharmaceuticals – but data and exposure remain limited compared with innovators. The regulation of biosimilars is a constantly evolving process, and the EMA has the most developed regulatory system for biosimilars. In the near future, the number of biosimilar medicines is likely to grow quite rapidly (Fig. 2), with several first-generation agents coming off-patent in the EU by 2014. In the oncology setting, we could see the development of biosimilar interferons and possibly, depending on regulatory developments, monoclonal antibodies such as anti-EGFR and anti-CD20. Regulatory processes will undoubtedly be refined and adapted as experience with biosimilar agents grows.

Biosimilars bring additional prescribing options; however, it is important for healthcare professionals to know the differences between these agents and a standard generic. Information on biosimilars remains limited, especially among oncologists and haematologists, and needs to be addressed in detail. In contrast to other biosimilars, in this therapeutic area products are given to immunosuppressed patients (who are at higher risk of complications) and to healthy stem cell donors (who derive no therapeutic benefit) thereby requiring the prescribing physician to have a more comprehensive knowledge of biosimilars.

The first step involves accurate naming of the product class and particularly the specific product, as some biosimilar agents have already been given the same INN as their reference product. Furthermore, extrapolation of indication leading to authorisation plays a major role, particularly in mobilisation procedures. In the next step, the product prescribed (innovator or biosimilar) should be defined and used during the whole treatment, which normally involves multiple cycles of therapy. Substitution (of innovator for biosimilar or between biosimilars) should be avoided as much as possible by describing brand name and INN to ensure traceability. While some EU countries already had regulations in place to prevent automatic substitution of medicinal products, many more have acted specifically to prevent the automatic substitution of biologics. Special attention should be given to labelling and the product SmPC, which sometimes provide information on the innovator (reference) product, rather than the biosimilar product itself. Reporting complications after treatment, especially long-term complications, becomes an important issue in patients treated with complex protocols and multiple lines of therapy. Physicians, pharmacists and patients should be aware of both the new possibilities and the new challenges posed by biosimilars.
